# Practice makes plasticity: 10-Hz rTMS enhances LTP-like plasticity in musicians and athletes

**DOI:** 10.3389/fncir.2023.1124221

**Published:** 2023-03-21

**Authors:** Jamie Kweon, Megan M. Vigne, Richard N. Jones, Linda L. Carpenter, Joshua C. Brown

**Affiliations:** ^1^Neuromodulation Research Facility, TMS Clinic, Butler Hospital, Providence, RI, United States; ^2^Department of Psychiatry and Human Behavior, Warren Alpert Medical School of Brown University, Providence, RI, United States; ^3^Department of Neurology, Warren Alpert Medical School of Brown University, Providence, RI, United States

**Keywords:** repetitive transcranial magnetic simulation, plasticity, LTP, motor-evoked potential (MEP), D-cycloserine (D-CYC: partial NMDA receptor agonist), NMDA-receptor

## Abstract

Motor skill learning has been linked to functional and structural changes in the brain. Musicians and athletes undergo intensive motor training through the practice of an instrument or sport and have demonstrated use-dependent plasticity that may be subserved by long-term potentiation (LTP) processes. We know less, however, about whether the brains of musicians and athletes respond to plasticity-inducing interventions, such as repetitive transcranial magnetic stimulation (rTMS), differently than those without extensive motor training. In a pharmaco-rTMS study, we evaluated motor cortex excitability before and after an rTMS protocol in combination with oral administration of D-cycloserine (DCS) or placebo. In a secondary covariate analysis, we compared results between self-identified musicians and athletes (M&As) and non-musicians and athletes (non-M&As). Three TMS measures of cortical physiology were used to evaluate plasticity. We found that M&As did not have higher baseline corticomotor excitability. However, a plasticity-inducing protocol (10-Hz rTMS in combination with DCS) strongly facilitated motor-evoked potentials (MEPs) in M&As, but only weakly in non-M&As. Placebo and rTMS produced modest facilitation in both groups. Our findings suggest that motor practice and learning create a neuronal environment more responsive to plasticity-inducing events, including rTMS. These findings may explain one factor contributing to the high inter-individual variability found with MEP data. Greater capacity for plasticity holds implications for learning paradigms, such as psychotherapy and rehabilitation, by facilitating LTP-like activation of key networks, including recovery from neurological/mental disorders.

## 1. Introduction

Motor skill training, defined as the acquisition and subsequent refinement of novel movement sequences in a progressive manner, has the ability to change the structure and function of the motor cortex (Adkins et al., 2006). Animal studies have found that training in a specific motor task produces an expansion of cortical representation of the exercised body part which parallels improved performance (reviewed in Adkins et al., 2006). In humans, 6 weeks of practice in a visuomotor task increased corticospinal excitability as measured by motor-evoked potentials (MEPs), with learning-dependent greater increases in those who engaged in increasingly difficult training (Christiansen et al., 2020). It is hypothesized that motor skill development on a short- and long-term scale can induce changes in synaptic strength between corticospinal neurons and engage synaptic plasticity to reorganize cortical maps (Monfils et al., 2005). Increased dendritic branching and synapse numbers have been found within the motor cortex of rats after motor skill training, suggesting learning-related synaptogenesis (Greenough et al., 1985; Kleim et al., 1996). Blocking receptors critical to synaptic plasticity abolishes practice-dependent effects at both cellular (animal) and behavioral (human) levels (see Monfils et al., 2005 for a comprehensive review).

Musicians and athletes (M&As) are two groups who engage in consistent, deliberate, motor skill acquisition through the practice of an instrument or sport. Studies have identified structural and functional differences in the brains of experienced M&As compared to non-M&As, specifically in regions engaged in consistent skill training (Herholz and Zatorre, 2012; Duru and Balcioglu, 2018). Pantev et al. (1998) showed brain responses to piano tones were 25% larger in musicians than in non-musicians, with larger effects for tones from each musician’s specific instrument. Another study found that skilled pianists demonstrated a rapid increase in motor cortex activation relative to non-musicians when performing a novel tapping task during a single fMRI scan (Hund-Georgiadis and von Cramon, 1999). Athletes also demonstrate this use-dependent plasticity phenomenon. One study using MRI found that golf experts engage less brain area than novices during a motor planning task, suggesting less recruitment necessary due to greater synaptic efficiency in skilled golfers (Milton et al., 2007). These changes in function are associated with changes in structure as evidenced by increased gray matter volume in hand areas of the motor cortex for handball players and increased gray matter volume in foot areas for ballet dancers (Meier et al., 2016). These results appear to suggest that motor learning maps onto generally associated regions of the motor cortex.

Transcranial magnetic stimulation (TMS) has been widely used to study motor cortex plasticity. 10-Hz repetitive TMS (rTMS) stimulation may induce long-term potentiation (LTP; Vlachos et al., 2012; Lenz et al., 2015; Brown et al., 2022), a form of synaptic plasticity which is critically dependent upon n-methyl-d-aspartate (NMDA) receptors (Huang et al., 2007). In humans, LTP-like processes can be assessed indirectly with quantitative neurophysiology and pharmacology capable of enhancing or diminishing key receptor activity while delivering rTMS. More specifically, 10-Hz rTMS to the motor cortex can increase MEP amplitudes (Maeda et al., 2000; Jung et al., 2008; Hoogendam et al., 2010) by enhancing LTP-like plasticity (Brown et al., 2020, 2021; Kweon et al., 2022). Plasticity induction is not unique to concurrent NMDA activation and 10-Hz rTMS, but has also been demonstrated with transcranial direct current stimulation (tDCS), transcranial alternating current stimulation (tACS), quadripulse stimulation (QPS), and continuous and intermittent theta burst stimulation (c/iTBS) including modified protocols [cTBS(mod)], to name a few, as reviewed in Brown et al. (2022) and Suppa et al. (2022). Importantly, MEPs utilize a corticomotor pathway to hand muscles that have been strengthened in M&As through motor skill training. M&As have demonstrated greater plasticity induction as measured by MEPs and recruitment (input-output) curves after paired-associative stimulation (PAS; Rosenkranz et al., 2007; Kumpulainen et al., 2015). We, therefore, hypothesized that subjects who routinely engaged in extensive motor learning and practice would have greater plasticity than those who did not. We present a secondary covariate analysis examining M&A status from a replication study (Kweon et al., 2022) designed to determine whether 10-Hz rTMS increases MEP amplitude through LTP-like mechanisms by assessing whether NMDA receptor agonism with d-cycloserine was sufficient to further enhance MEPs, as shown previously (Brown et al., 2020).

## 2. Materials and methods

**Figure 1:**
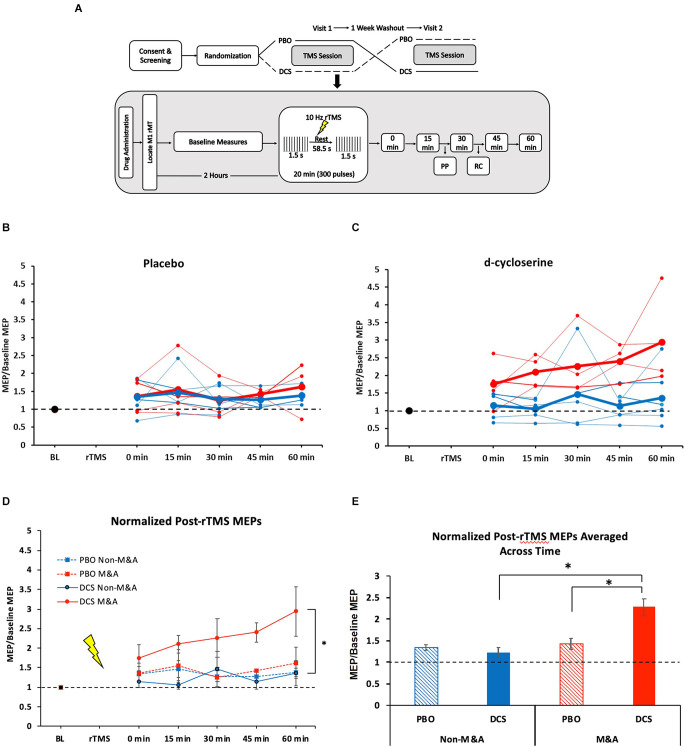
NMDA receptor partial agonist D-cycloserine (DCS) enhances 10-Hz rTMS-induced motor-evoked potentials (MEPs) exclusively for Musicians and Athletes. **(A)** Study design. Top: Overview of full experiment. Below: TMS session protocol. Baseline measures include SP, PP, and RC. MT and SP bins were recorded at every 15-min post-rTMS time points. **(B)** Individual subject MEP values for each time point after 10-Hz rTMS for placebo condition. Blue = Non-M&As, Red = M&As. Group averages are in bold. **(C)** Individual subject MEP values for each time point after 10-Hz rTMS for d-cycloserine condition. Blue = Non-M&As, Red = M&As. Group averages are in bold. **(D)** Averaged (normalized to baseline) MEP values with standard error of the mean (error bars) for each time point after 10-Hz rTMS for all conditions: 0 min: Non-M&As (1.15 ± 0.14), M&As (1.75 ± 0.34), *H*_(3)_ = 3.06, *p* = 0.383; 15 min: Non-M&As (1.06 ± 0.11), M&As (2.1 ± 0.23), *H*_(3)_ = 7.69, *p* = 0.053; 30 min: Non-M&As (1.47 ± 0.45), M&As (2.26 ± 0.49), *H*_(3)_ = 4.93, *p* = 0.024; 45 min: Non-M&As (1.14 ± 0.18), M&As (2.4 ± 0.24), *H*_(3)_ = 9.48, *p* = 0.024; 60 min: Non-M&As (1.37 ± 0.32), M&As (2.94 ± 0.64), *H*_(3)_ = 6.70, *p* = 0.082. **(E)** Normalized MEP values averaged across time with standard error of the mean (error bars) for all conditions: PBO Non-M&As (1.35 ± 0.07), DCS Non-M&As (1.23 ± 0.12), PBO M&As (1.43 ± 0.12), DCS M&As (2.29 ± 0.019). ^*^p < 0.05. PBO, Placebo; DCS, D-cycloserine; SP, single-pulse; PP, paired-pulse; RC, recruitment curve; rMT, resting motor threshold; rTMS, repetitive Transcranial Magnetic Stimulation; MEPs, motor-evoked potentials; M&As, Musicians and Athletes; ICF, intracortical facilitation.

We analyzed results from 10 healthy adults (six women, 21–39 years old) from a randomized, double-blind, crossover study, as described previously (Kweon et al., 2022). In that study, subjects received a single dose of either 100 mg dose of D-cycloserine (DCS, an NMDA receptor partial agonist) or placebo, then the other capsule, in random order, at least 1 week apart. We collected baseline MEP (bins of 20 single- or paired-pulse jittered 4–7 s apart) measures ~1 h after dosing, followed by the rTMS plasticity protocol ~2 h after dosing, and finally, post-rTMS MEP measures, as shown in Figure 1A. All subjects were right-handed.

All TMS single-pulses and rTMS pulse trains were delivered with the PowerMag stimulator system (Mag and More, Germany). Briefly, 10-Hz rTMS was delivered at 80% of the resting motor threshold in 1.5 s trains with 58.5 s rest for 20 min (300 pulses). All pulses were neuronavigated within 0.5 mm of the left motor cortex (M1) (Brainsight, Rogue Research, Quebec, Canada). Procedures for obtaining resting motor threshold (rMT) were described in the original study (Kweon et al., 2022). We collected one bin of 40 single-pulses (SP) at 120% rMT, and one SP at every percent intensity from 20% to 100% of maximum machine stimulator output in randomized order fit to a Boltzmann sigmoidal recruitment curve (RC). Pulses were jittered at 4–7 s intervals. Paired pulses were separated by inter-stimulus interval of 3 ms for SICI and 15 ms for ICF conditioning stimulus (CS), with a subthreshold intensity (80% rMT) and the testing stimulus (TS) of 120% rMT. LICI consisted of two pulses at 120% rMT spaced 100 ms apart.

As a part of our demographic questionnaire, we asked participants if they were an “experienced musician or athlete” who currently practiced said skill. Those who selected “yes” were asked to record the number of years and hours per week they spent practicing. Four of the 10 participants identified as a musician or athlete, with an average length of practice of 14.5 years, and a range of 4–6 h per week (Table 1).

**Table 1:** Characteristics of musicians/athletes.

Musician/Athlete	Male (M)/Female (F)	Age (years)	Instrument/Sport	Number of Years	Practice intensity (hours/week)
1	F	39	Piano	33	4
2	F	35	Piano	8	4.5
3	F	28	Guitar	4	4.5
4	F	22	Volleyball	14	6

As before, we analyzed SP data over a 1-h time course, normalized to baseline, using a mixed repeated measure analysis of variance (ANOVA). We examined the effects of drug, time, and drug-time interaction, controlling for order in the crossover design. Given the small sample size, we could not assume normality; therefore, a Kruskal-Wallis test was used to compare differences at baseline and each post-rTMS time point between the four drug (DCS, placebo) × group (M&A, non-M&A) conditions. The overall effect was also analyzed with a Kruskal-Wallis test of the grand average of all post-rTMS MEP amplitudes (across time) between the four conditions. Paired-pulse (PP) measures were derived from a ratio of PP/SP, and these ratios were compared before and after rTMS to generate a percent change, as described previously (Brown et al., 2021). The Kruskal-Wallis test was used to compare averages across the four conditions. Mann-Whitney U test was used to analyze characteristics between groups and visits (i.e., age, motor threshold). Recruitment curves were fitted with the Levenberg-Marquard nonlinear least-mean squares algorithm to fit raw data to a Boltzmann sigmoidal function using Signal software (Cambridge Electronic Devices, UK) as before (Kweon et al., 2022). Wilcoxon signed-rank test compared the change in recruitment curve intercept, slope, and height before and after rTMS and between drug conditions. Analyses were performed with R software (R core team, Vienna, Austria). We set *a priori* level of significance at *p* < 0.05.

## 3. Results

We found no differences between Non-M&As and M&As regarding age [U (N_Non-M&As_ = 6, N_M&As_ = 4) = 7.5, *p* = 0.39] or resting motor threshold (rMT) averaged across the two visits [U (N_Non-M&As_ = 6, N_M&As_ = 4) = 32, *p* = 0.23]. We also found no difference in baseline MEPs between Non-M&As and M&As in either drug condition (Kruskal-Wallis, *H*_(3)_ = 7.02, *p* = 0.07; Supplementary Figure 1A).

### 3.1. Single pulse time course

Figures 1B,C displays individual subject MEP values for each time point after 10-Hz rTMS by drug condition. In the placebo condition, M&As did not differ from non-M&As by group (*F*_(1,4)_ = 0.87, *p* = 0.404), time (*F*_(4,16)_ = 0.53, *p* = 0.713), or interaction (*F*_(4,16)_ = 1.93, *p* = 0.155), as shown in Figure 1D. There was a group effect when taking DCS, however, with M&As having greater normalized MEP amplitudes repeated across time compared with Non-M&As (*F*_(1,4)_ = 7.8, *p* = 0.027, *η*^2^ = 0.40; Figure 1D), without a definitive effect of time (*F*_(4,16)_ = 2.5, *p* = 0.06) or group-by-time interaction (*F*_(4,16)_ = 0.97, *p* = 0.44).

Grand averages of all normalized time points across the four conditions yielded a marked increase for M&A with DCS (*H*_(3)_ = 29.23, *p* < 0.001; Figure 1E), including direct comparisons between drug conditions for M&As [*U* (N_PBO_ = 19, N_DCS_ = 20) = 63, *z* = −3.57, *p* < 0.001], and between M&As and non-M&As within the DCS condition [*U* (N_Non-M&As_ = 29, N_M&As_ = 20) = 62, *Z* = −4.64, *p* < 0.001].

### 3.2. Paired pulse

We did not detect a difference with our small sample size in the degree of ICF change before and after rTMS between the four combinations of drug × group (*H*_(2)_ = 1.079, *p* = 0.583, Supplementary Figures 2A,D). We also detected no differences in SICI measures (*H*_(3)_ = 1.541, *p* = 0.673, Supplementary Figures 2B,E), or LICI measures (*H*_(3)_ = 1.512, *p* = 0.680, Supplementary Figures 2C,F).

### 3.3. Recruitment curve

We did not detect any differences in recruitment curve intercepts, slopes, or heights either between conditions or before and after rTMS. We did find a trend-level decrease in intercept after rTMS for M&As in the DCS condition (*z* = −1.826, *p* = 0.068, Supplementary Figure 3).

## 4. Discussion

These data appear to be consistent with our hypothesis that motor learning can enhance the capacity to respond to plasticity-inducing events. Specifically, we observed that 10-Hz rTMS + d-cycloserine robustly increased MEPs over 1-h for M&As. We also observed that trend level increases in intracortical facilitation and excitatory shifts in recruitment curves. However, it is important to note that these results are from a post-hoc covariate analysis from a small sample size of 10 subjects (20 visits), and therefore we cannot reach conclusions, but we present these data to assist in future study design. This limitation acknowledged, we speculate on what these results, if true, might mean with implications for repetitious practice and plasticity (i.e., rTMS, psychotherapy, rehabilitation, etc.).

DCS has previously been shown to enhance the excitatory effects of 10-Hz rTMS on MEPs in healthy participants (Brown et al., 2020; Kweon et al., 2022), purportedly through NMDA receptor agonism. LTP requires NMDA receptor activity (Brown et al., 2022), as do the LTP-like changes found in animal hippocampal slices after 10-Hz repetitive magnetic stimulation (Vlachos et al., 2012). More specifically, LTP (and learning) can be enhanced by increasing NMDA activity, as demonstrated in transgenic mice overexpressing an NMDA receptor subunit (Tang et al., 1999, 2001). Thus, the enhancement of 10-Hz rTMS-induced MEPs through d-cycloserine augmentation may reasonably be thought to enact LTP-like mechanisms by associating NMDA receptor activation with neuronal stimulation. Extensive motor practice in M&As incorporates the long-term effects of increased learning, which is known to be subserved by NMDA-receptor dependent LTP (Whitlock et al., 2006; Brown et al., 2021). The applicability of these results outside of the motor cortex and healthy subjects has been supported by Cole and colleagues who found that DCS was sufficient to enhance both motor physiology (Cole et al., 2021), and clinical outcomes in depressed patients (Cole et al., 2022).

Interestingly, baseline MEPs for M&As showed a trend-level decrease (see Supplementary Figure 1A). If true, this would suggest that M&As do not have higher baseline corticomotor excitability, but rather with *a greater capacity to undergo change*, the definition of plasticity (Brown et al., 2022). To speculate further on the molecular mechanism of these changes, we can consider what is known from animal studies. Baseline excitability (synaptic transmission) is mediated primarily by α-amino-3-hydroxy-5-methyl-4-isoxazolepropionic acid (AMPA) receptors (Muller et al., 1988). Increased excitability is mediated by an acute increase in the GluA1 subtype of AMPA receptors (Brown et al., 2022) which has already been demonstrated to occur with 10-Hz magnetic stimulation (Vlachos et al., 2012) and learning (Whitlock et al., 2006). NMDA receptors, on the other hand, are not important for baseline transmission but are critical for governing AMPA receptor trafficking into the synapse, that is, LTP (Brown et al., 2021). If we extrapolate these animal-level findings to M&As, it may be that M&As have less M1 AMPA receptors at baseline, but can quickly upregulate AMPA receptors possibly as a result of increased NMDA receptors. This appears plausible given that LTP upregulates NMDA receptor expression, and that this is necessary for subsequent learning (Yang et al., 2022). Increased NMDA receptors in M&As would indicate a greater capacity to induce LTP with appropriate synaptic activation.

In promoting plasticity induction, it is often tempting to apply the expression “more is better”. However, with non-invasive brain stimulation, we often see this is not the case. Rather, an inverted U-shape curve indicates a “sweet spot” for much of plasticity induction (Caulfield and Brown, 2022). In fact, in many cases, more stimulation may reverse results, such as by doubling iTBS pulse numbers (Gamboa et al., 2010). It is not yet known whether protocols involving DCS could be further enhanced. We administered a single session of 300 pulses. It remains to be seen whether plasticity induction could be further increased with clinical protocols involving 3,000 pulses for 36 sessions, or if mechanisms would be invoked and effects reversed (Thomson and Sack, 2020). This may explain the long-term depression (LTD)-like effects seen with 600 pulses of iTBS with DCS (Teo et al., 2007). While this would seem counterintuitive in the acute sense, chronic learning or repeated LTP would engage homeostatic plasticity mechanisms which effectively serve to prevent ceiling effects (saturation) and enable continuous learning through whole-neuron AMPA receptor expression reduction while still retaining relative synaptic strength (Turrigiano, 2012). Homeostatic metaplasticity may also explain our observation that M&As trended towards lower baseline MEPs, theoretically reflecting a decrease in AMPA receptors, but an increase in NMDA receptors (Yang et al., 2022), which do not mediate most of baseline synaptic transmission (Muller et al., 1988). Whether homeostatic AMPA receptor removal and LTP-induced NMDA receptor upregulation is independent of one another, or possibly connected in the same manner that GluA2 receptors replace GluA1 receptors (Shi et al., 2001) is an intriguing question. Regardless, if MEP excitability is mediated by AMPA receptors, these results appear consistent with M&As having decreased AMPA receptors at baseline, while the marked increase as a result of a plasticity protocol may be orchestrated by increased NMDA receptors from mature synapses.

These data provide preliminary support that repeated learning (as experienced by M&As) may enhance synaptic plasticity (Rosenkranz et al., 2007; Kumpulainen et al., 2015) induced by 10-Hz rTMS and NMDA receptor activation. However, a larger sample size is necessary to reach conclusions, and a prospective study design (examining before and after musical/athletic training) would be needed to determine causality (i.e., whether increased plasticity is produced by learning or whether people with innately enhanced plasticity are more likely to become M&As). Furthermore, our sample included three types of instruments and sports, which likely activate plasticity in various brain regions to different extents. This is both a limitation of our *post-hoc* naturalistic study design as well as valuable information regarding the generalizability of motor practices on specific muscle groups. Future studies may determine how generalizable these changes are by comparing, for example, pianists and violinists. We have speculated about underlying mechanisms which can only be guessed at without parallel human and animal experimental designs. M&As may have driven previously reported differences following 10-Hz rTMS (Brown et al., 2020), and may contribute to the high inter-individual variability frequently found in MEP studies (Corp et al., 2020, 2021). Of broader interest is whether motor network learning translates to other networks, such as those theoretically targeted with dorsolateral prefrontal cortex rTMS for depression. It is tempting to consider whether prior extensive application of psychotherapeutic techniques (enhancing plasticity capacity of relevant circuits) promotes responsiveness to clinical rTMS, or whether rTMS alone accomplishes this in responders. These preliminary results suggest that learning may facilitate LTP-like activity in relevant neural circuits with implications for therapeutic contexts like stroke rehabilitation, cognitive-behavioral therapy, or clinical rTMS.

## Data availability statement

The raw data supporting the conclusions of this article will be made available by the authors, without undue reservation.

## Ethics statement

The studies involving human participants were reviewed and approved by Care New England-Butler Hospital Institutional Review Board. The patients/participants provided their written informed consent to participate in this study.

## Author contributions

JK collected data and wrote the manuscript. MV collected data and significantly contributed to the manuscript. RJ provided statistics support and manuscript edits. JB as principal investigator and head of lab, significantly contributed to manuscript. LC as head of the transcranial magnetic stimulation clinic provided equipment and resources as well as manuscript edits. All authors contributed to the article and approved the submitted version.
