# Supplementary material for: Practice makes plasticity: 10-Hz rTMS enhances LTP-like plasticity in musicians and athletes
Source: Front Neural Circuits. 2023 Mar 21;17:1124221. doi: 10.3389/fncir.2023.1124221 (PMC10070804; doi:10.3389/fncir.2023.1124221)
Supplement: Supplementary file 1 [file Data_Sheet_1.docx]

Supplementary Material

**Practice Makes Plasticity: 10-Hz rTMS Enhances LTP-like Plasticity in Musicians and Athletes**

Jamie Kweon^1^, Megan M. Vigne^1^, Rich N. Jones^2.3^, Linda L. Carpenter^1,2^, Joshua C. Brown^1,2.3^*

^1^ Neuromodulation Research Facility, TMS Clinic, Butler Hospital, Providence, RI, United States

^2^ Department of Psychiatry and Human Behavior, Warren Alpert Medical School of Brown University, Providence, RI, United States

^3^ Department of Neurology, Warren Alpert Medical School of Brown University, Providence, RI, United States

*** Correspondence:**Joshua Brown
joshua_c_brown@brown.edu

# Supplementary Figures and Tables

Supplementary Materials


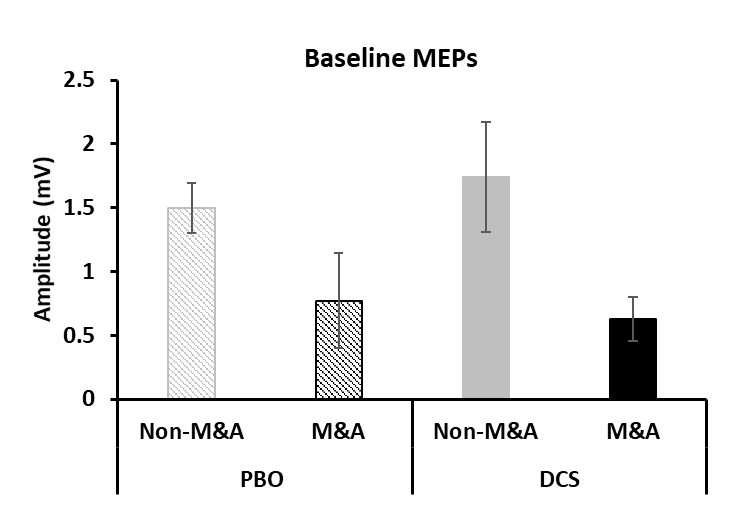


**Supplementary Figure 1.** Baseline MEPs with standard error of the mean (error bars) for all conditions. PBO Non-M&As (1.50±0.19), PBO M&As (0.772±0.37), DCS Non-M&As (1.74±0.43), DCS M&As (0.627±0.17).

1. **B. C.**

**
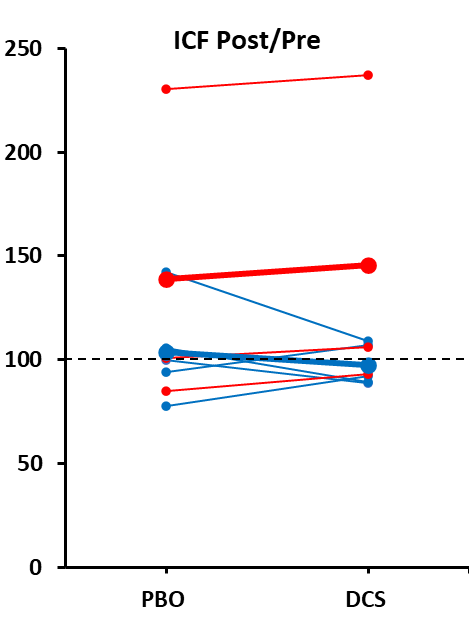

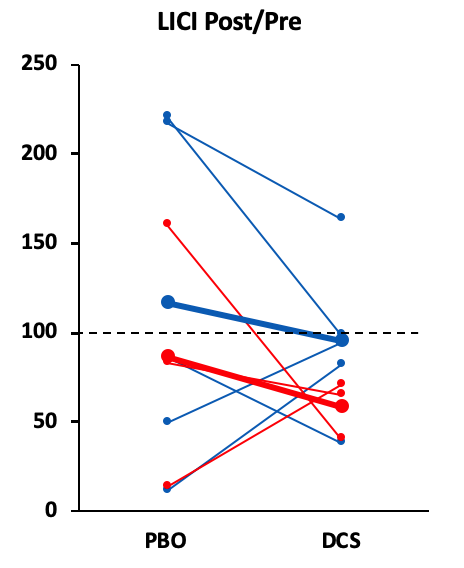
**
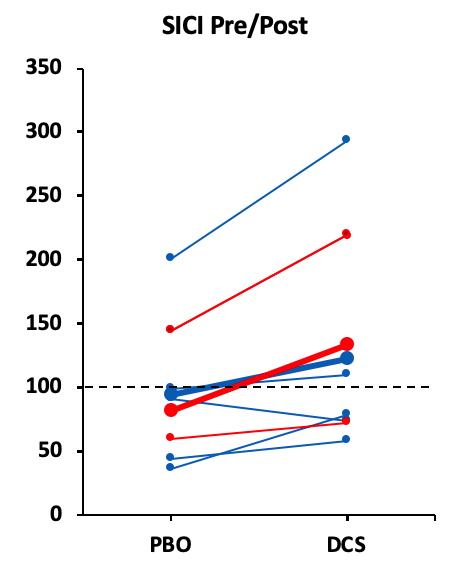


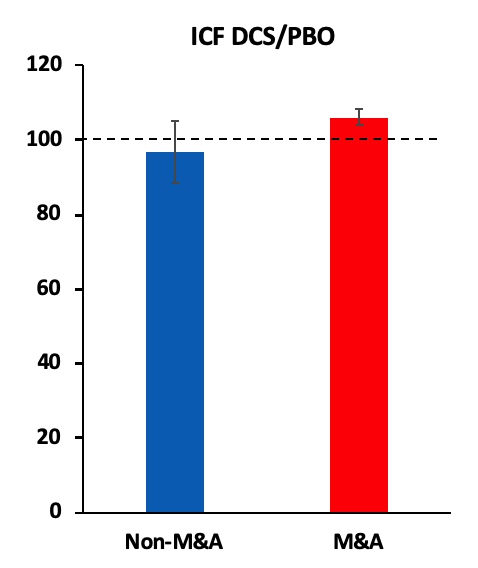
**D. E. F.**

**
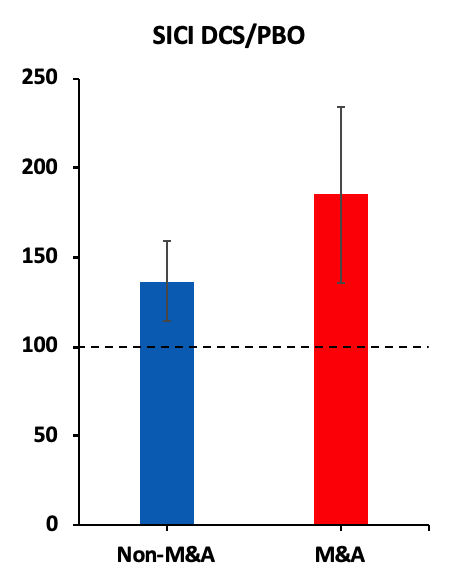

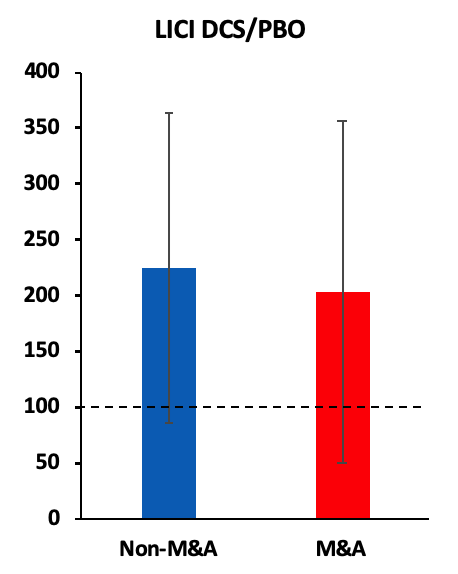
**

**Supplementary Fig. 2.** **(A-C)** Individual data for percent change after 10-Hz rTMS by drug condition. Blue= Non-M&A, Red= M&A (Group averages in bold): **(A)** ICF: PBO Non-M&As (103.9), DCS Non-M&As (97.36), PBO M&As (138.7), DCS M&As (145.43). **(B)** SICI: PBO Non-M&As (94.35), DCS Non-M&As (122.4), PBO M&As (80.79), DCS M&As (133.22). (**C)** LICI: PBO Non-M&As (116.5), DCS Non-M&As (95.10), PBO M&As (85.71), DCS M&As (58.16). **(D-F)** Average percent change between drug conditions by M&A status: **(D)** Non-M&A (96.57±8.23), M&A (105.9±2.67), p=.92. **(E)** SICI: Non-M&A (136.4±22.3), M&A (184.7±49.4), p=.44. (**F)** LICI: Non-M&A (225.1±139), M&A (203.2±153), p=.32. Error bars=standard error of the mean. PBO= Placebo; DCS= D-cycloserine; rTMS= repetitive Transcranial Magnetic Stimulation; MEPs= motor-evoked potentials; M&As= Musicians and Athletes; ICF= intracortical facilitation; SICI= short intracortical inhibition; LICI= long intracortical inhibition.

**A. B.** **
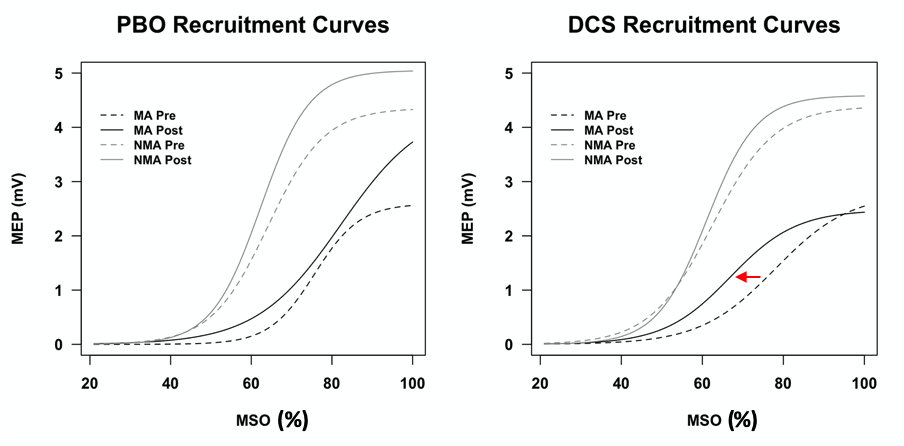
**


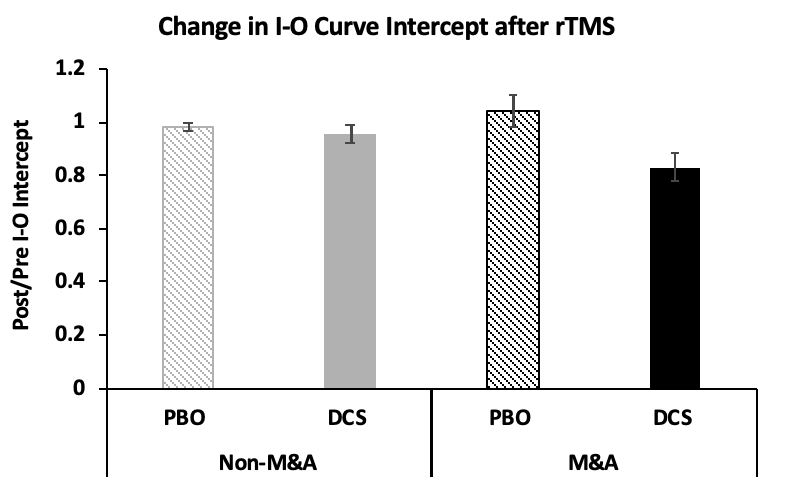


**C.**

**Supplemental Fig. 3. (A)** Recruitment curves generated for Non-M&As and M&As before and after rTMS in PBO condition and **(B)** DCS condition **(C)** Change in X_50_ from Pre- to Post-rTMS across all conditions. Decrease in intercept for M&As before to after rTMS in DCS condition (p=.068). PBO Non-M&As (0.98±0.02), DCS Non-M&As (0.96±0.03), PBO M&As (1.05±0.05), DCS M&As (0.84±0.05).
